# Habitat-dependent *Culicoides* species composition and abundance in blue tit (*Cyanistes caeruleus*) nests

**DOI:** 10.1017/S003118202200066X

**Published:** 2022-07

**Authors:** Jorge Garrido-Bautista, Josué Martínez-de la Puente, José Luis Ros-Santaella, Eliana Pintus, Paula Lopezosa, Nicola Bernardo, Mar Comas, Gregorio Moreno-Rueda

**Affiliations:** 1 Department of Zoology, Faculty of Sciences, University of Granada, 18071 Granada, Spain; 2 Department of Parasitology, Faculty of Pharmacy, University of Granada, 18071 Granada, Spain; 3 CIBER of Epidemiology and Public Health (CIBERESP), Madrid, Spain; 4 Department of Veterinary Sciences, Faculty of Agrobiology, Food and Natural Resources, Czech University of Life Sciences Prague, Kamýcká 129, 16500 Prague 6-Suchdol, Czech Republic; 5 Department of Ecology, University of Alicante, 03690 Alicante, Spain; 6 Biological Station of Doñana, EBD-CSIC, Av. Américo Vespucio 26, 41092 Seville, Spain; 7 Department of Biological Sciences, Dartmouth College, Hanover, NH 03755, USA

**Keywords:** Avian malaria, avian nests, biting midges, blood-feeding insects, *Haemoproteus*, host selection, vectors

## Abstract

Wild birds are hosts of *Culicoides* from as early on as the nesting stage when constrained to their nests. However, the environmental factors which determine the abundance and composition of *Culicoides* species within each bird nest are still understudied. We sampled *Culicoides* from Eurasian blue tit (*Cyanistes caeruleus*) nests found in 2 types of forests located in southern Spain. Firstly, we monitored the abundance of *Culicoides* species in bird nests from a dry Pyrenean oak deciduous forest and a humid mixed forest comprising Pyrenean and Holm oaks throughout 2 consecutive years. During the 3rd year, we performed a cross-fostering experiment between synchronous nests to differentiate the role of rearing environment conditions from that of the genetically determined or maternally transmitted cues released by nestlings from each forest. We found 147 female *Culicoides* from 5 different species in the birds' nests. The abundance of *Culicoides* was higher in the dry forest than in the humid forest. *Culicoides* abundance, species richness and prevalence were greater when the nestlings were hatched later in the season. The same pattern was observed in the cross-fostering experiment, but we did not find evidence that nestling's features determined by the forest of origin had any effect on the *Culicoides* collected. These results support the notion that habitat type has a strong influence on the *Culicoides* affecting birds in their nests, while some life history traits of birds, such as the timing of reproduction, also influence *Culicoides* abundance and species composition.

## Graphical abstract

**Figure:**
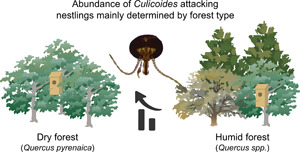

## Introduction

*Culicoides* biting midges (Diptera: Ceratopogonidae) are one of the world's smallest and most abundant blood-sucking flies (Mellor *et al*., 2000). *Culicoides* have a widespread distribution with haematophagous females acting as vectors of various pathogens. Most research on this group focuses on its role in the transmission of viruses (e.g. African horse sickness virus, bluetongue virus or Schmallenberg virus, among others) to livestock, because of its economic impact on the industry (Mellor *et al*., 2000; Carpenter *et al*., 2013; Sick *et al*., 2019; Martínez-de la Puente *et al*., 2021). However, *Culicoides* are well-known vectors of other parasites including avian trypanosomes (Svobodová *et al*., 2017) and the avian malaria-like *Haemoproteus* (Valkiūnas, 2005; Martínez-de la Puente *et al*., 2011*a*), also supporting their role in the transmission of parasites to wildlife. In spite of this, there are still relatively few studies into the ecological interactions between biting midges and wild birds. This is especially relevant considering the impact of *Culicoides* on the body condition of nestlings (Tomás *et al*., 2008*a*), together with the deleterious effects of the *Culicoides*-borne parasites on bird health (Merino *et al*., 2000; Tomás *et al*., 2008*a*; Martínez-de la Puente *et al*., 2010*a*). The development of traps inside nest boxes (Tomás *et al*., 2008*b*; Votýpka *et al*., 2009) allowed researchers to identify the diversity of *Culicoides* species attracted to bird nests in different European regions (Table 1). For instance, Martínez-de la Puente *et al*. (2009*a*) identified the presence of 7 different species of *Culicoides* in blue tit (*Cyanistes caeruleus*) nests found in central Spain. Additional studies have also identified which species of *Culicoides* are attacking birds in their nests, including studies on different avian species conducted in several countries (Czech Republic: Votýpka *et al*., 2009; Spain: Veiga *et al*., 2018; Lithuania and Russia: Žiegytė *et al*., 2021). Moreover, the development of molecular techniques to identify the origin of blood meals from engorged biting midge females has confirmed the ornithophilic feeding preference of most *Culicoides* species collected from bird nests (Bobeva *et al*., 2015; Martínez-de la Puente *et al*., 2015). Thus, the results from these articles support the fact that different species of biting midges are attracted to bird nests, but there is still a lack of studies into what determines *Culicoides* species composition and abundance.

**Table 1.** Summary of *Culicoides* species found in nest boxes of different European bird species, namely blue tit (*Cyanistes caeruleus*), great tit (*Parus major*), pied flycatcher (*Ficedula hypoleuca*) and European roller (*Coracias garrulus*)

*Culicoides* species	Bird host^a^	Region	Country	*Haemoproteus* positive	Reference
*Culicoides circumscriptus*	Blue tit	Granada	Spain	Not examined	This study
		Segovia	Spain	Not examined	Martínez-de la Puente *et al*. (2009*a*)
		Segovia	Spain	Yes	Martínez-de la Puente *et al*. (2011*a*)
	European roller	Almería	Spain	Yes	Veiga *et al*. (2018)
	Not reported	Mikulov	Czech Republic	Not examined	Votýpka *et al*. (2009)
*Culicoides duddingstoni*	Not reported	Mikulov	Czech Republic	Not examined	Votýpka *et al*. (2009)
*Culicoides festivipennis*	Blue tit	Granada	Spain	Not examined	This study
		Segovia	Spain	Not examined	Martínez-de la Puente *et al*. (2009*a*)
		Segovia	Spain	Yes	Martínez-de la Puente *et al*. (2011*a*)
	Great tit	Vilnius	Lithuania	No	Žiegytė *et al*. (2021)
		Rybachy	Russia	No	Žiegytė *et al*. (2021)
	Pied flycatcher	Vilnius	Lithuania	No	Žiegytė *et al*. (2021)
		Rybachy	Russia	No	Žiegytė *et al*. (2021)
	Not reported	Mikulov	Czech Republic	Not examined	Votýpka *et al*. (2009)
*Culicoides impunctatus*	Great tit	Vilnius	Lithuania	No	Žiegytė *et al*. (2021)
		Rybachy	Russia	No	Žiegytė *et al*. (2021)
	Pied flycatcher	Vilnius	Lithuania	No	Žiegytė *et al*. (2021)
		Rybachy	Russia	No	Žiegytė *et al*. (2021)
*Culicoides kibunensis*	Blue tit	Granada	Spain	Not examined	This study
		Segovia	Spain	Not examined	Martínez-de la Puente *et al*. (2009*a*)
		Segovia	Spain	Yes	Martínez-de la Puente *et al*. (2011*a*)
	Great tit	Vilnius	Lithuania	No	Žiegytė *et al*. (2021)
		Rybachy	Russia	Yes	Žiegytė *et al*. (2021)
	Pied flycatcher	Vilnius	Lithuania	No	Žiegytė *et al*. (2021)
		Rybachy	Russia	Yes	Žiegytė *et al*. (2021)
	Not reported	Mikulov	Czech Republic	Not examined	Votýpka *et al*. (2009)
*Culicoides minutissimus*	Not reported	Mikulov	Czech Republic	Not examined	Votýpka *et al*. (2009)
*Culicoides obsoletus* complex	Great tit	Vilnius	Lithuania	No	Žiegytė *et al*. (2021)
		Rybachy	Russia	No	Žiegytė *et al*. (2021)
	Pied flycatcher	Vilnius	Lithuania	No	Žiegytė *et al*. (2021)
		Rybachy	Russia	No	Žiegytė *et al*. (2021)
*Culicoides pallidicornis*	Great tit	Rybachy	Russia	No	Žiegytė *et al*. (2021)
	Pied flycatcher	Rybachy	Russia	No	Žiegytė *et al*. (2021)
*Culicoides paolae*	European roller	Almería	Spain	Yes	Veiga *et al*. (2018)
*Culicoides pictipennis*	Blue tit	Segovia	Spain	Not examined	Martínez-de la Puente *et al*. (2009*a*)
		Segovia	Spain	No	Martínez-de la Puente *et al*. (2011*a*)
	Great tit	Vilnius	Lithuania	No	Žiegytė *et al*. (2021)
		Rybachy	Russia	Yes	Žiegytė *et al*. (2021)
	Pied flycatcher	Vilnius	Lithuania	No	Žiegytė *et al*. (2021)
		Rybachy	Russia	Yes	Žiegytė *et al*. (2021)
	Not reported	Mikulov	Czech Republic	Not examined	Votýpka *et al*. (2009)
*Culicoides punctatus*	Great tit	Rybachy	Russia	Yes	Žiegytė *et al*. (2021)
	Pied flycatcher	Rybachy	Russia	Yes	Žiegytė *et al*. (2021)
*Culicoides reconditus*	Blue tit	Granada	Spain	Not examined	This study
	Great tit	Vilnius	Lithuania	No	Žiegytė *et al*. (2021)
		Rybachy	Russia	No	Žiegytė *et al*. (2021)
	Pied flycatcher	Vilnius	Lithuania	No	Žiegytė *et al*. (2021)
		Rybachy	Russia	No	Žiegytė *et al*. (2021)
*Culicoides segnis*	Blue tit	Segovia	Spain	Not examined	Martínez-de la Puente *et al*. (2009*a*)
		Segovia	Spain	Yes	Martínez-de la Puente *et al*. (2011*a*)
	Great tit	Vilnius	Lithuania	Yes	Žiegytė *et al*. (2021)
		Rybachy	Russia	Yes	Žiegytė *et al*. (2021)
	Pied flycatcher	Vilnius	Lithuania	Yes	Žiegytė *et al*. (2021)
		Rybachy	Russia	Yes	Žiegytė *et al*. (2021)
*Culicoides simulator*	Blue tit	Segovia	Spain	Not examined	Martínez-de la Puente *et al*. (2009*a*)
		Segovia	Spain	Yes	Martínez-de la Puente *et al*. (2011*a*)
	Not reported	Mikulov	Czech Republic	Not examined	Votýpka *et al*. (2009)
*Culicoides subfascipennis*	Great tit	Vilnius	Lithuania	No	Žiegytė *et al*. (2021)
	Pied flycatcher	Vilnius	Lithuania	No	Žiegytė *et al*. (2021)
*Culicoides sphagnumensis*	Great tit	Rybachy	Russia	No	Žiegytė *et al*. (2021)
	Pied flycatcher	Rybachy	Russia	No	Žiegytė *et al*. (2021)
*Culicoides truncorum*	Blue tit	Granada	Spain	Not examined	This study
		Segovia	Spain	Not examined	Martínez-de la Puente *et al*. (2009*a*)
		Segovia	Spain	Yes	Martínez-de la Puente *et al*. (2011*a*)
	Not reported	Mikulov	Czech Republic	Not examined	Votýpka *et al*. (2009)

a Votýpka *et al*. (2009) sampled biting midges from the nests of tree sparrow (*Passer montanus*), great tit (*P. major*), blue tit (*C. caeruleus*), spotted flycatcher (*Muscicapa striata*), nuthatch (*Sitta europaea*) and wryneck (*Jynx torquilla*).

The type of habitat may have a significant effect on the *Culicoides* species breeding in the area, ultimately determining the abundance and composition of *Culicoides* inside the bird nests. The type of substrate and water sources are essential for the development of *Culicoides* larvae (Uslu and Dik, 2010; Erram *et al*., 2019), but the distance to water sources from avian nests does not seem to affect the abundance of adult biting midges in the nests (Tomás *et al*., 2008*b*). In addition, weather conditions, which are partially modulated by habitat characteristics, may also influence the abundance of biting midges in avian nests. For example, higher winds early in the morning may negatively affect *Culicoides* flight performance and, consequently, reduce their abundance in nests (Martínez-de la Puente *et al*., 2009*b*). Nevertheless, there is scant information about how habitat-dependent variation in environmental factors may affect *Culicoides* abundance and species composition inside nests. In fact, only a few studies have examined the spatial variation of biting midge abundance in natural habitats. For instance, the management and structure of the habitat may affect the abundance of *Culicoides* in the area (van Hoesel *et al*., 2019), with more *Culicoides* females captured in the canopy compared to the ground level in a vertical axis (Černý *et al*., 2011). *Culicoides* abundance may also vary between habitats, but this depends on the host breeding period (Tomás *et al*., 2020) and patch extension (Rivero de Aguilar *et al*., 2018). Several factors related to avian host physiology, behaviour and breeding performance may also affect the number of biting midges entering nests. Haematophagous vectors such as *Culicoides* use different cues (e.g. odourant molecules) to locate their hosts, such as 1-octen-3-ol, carbon dioxide (CO_2_) and kairomones (Bhasin *et al*., 2000*a*, 2000*b*; Castaño-Vázquez *et al*., 2020). This may at least partly explain the positive correlation between *Culicoides* abundance and brood size in different bird species (Martínez-de la Puente *et al*., 2009*a*, 2009*b*; Martínez-de la Puente *et al*., 2010*b*; Castaño-Vázquez and Merino, 2021), likely due to a greater release of attractive molecules from nests with larger broods.

The aim of this study was to identify the effect of habitat type on the abundance and species composition of biting midge *Culicoides* attacking avian hosts in their nests. To this end, we sampled *Culicoides* in nest boxes occupied by blue tits in 2 neighbouring forests in southern Spain with different environmental characteristics during the bird's breeding season for 3 consecutive years. We first compared the prevalence, abundance and species richness in nests situated in the 2 habitat types over the first 2 years. Secondly, we developed a cross-fostering experiment based on the results obtained and considering that the observed differences between the 2 forests could be due to the effects of habitat type on the emission of nestling cues (e.g. microbiota, Ruiz-López, 2020; or the composition of uropygial secretions, Tomás *et al*., 2020), rather than a direct association between habitat type and *Culicoides*. This study design meant we could examine the role of rearing environment conditions (i.e. forest type) independently from the genetically determined attractants released by birds in each forest.

## Materials and methods

### Study area

The study was carried out during the spring of 2017, 2018 and 2019 using blue tits breeding in nest boxes in the Sierra Nevada National Park (southeast Spain, 36°57′N, 3°24′W, 1700–1800 m a.s.l.). Nest boxes were placed in 2 different, but adjacent forests separated by approximately 1.5 km. One site was a dry Pyrenean oak (*Quercus pyrenaica*) deciduous forest (hereinafter, ‘dry forest’), while the other was a mixed forest consisting mainly of Pyrenean oaks along with some Holm oaks (*Quercus ilex*) and which was crossed by a stream (Acequia Almiar), conferring it a moister ambient (hereinafter, ‘humid forest’). The humid forest had a higher relative humidity, lower mean temperature, higher solar irradiation and lower insolation time compared to the dry forest (see Supplementary material; Garrido-Bautista *et al*., 2021).

### Blue tit sampling and cross-fostering experiment

Overall, 199 nest boxes were occupied by blue tits over the 3 years (69 in 2017, 56 in 2018 and 74 in 2019), of which 95 were randomly selected and followed to collect the data included in this study. Biting midges were monitored in 45 nest boxes in the dry forest and 50 in the humid forest. All the nest boxes were ICONA C model (details in Moreno-Rueda, 2003) and were cleaned every year before the breeding season. They were hung from an oak tree's branch at a height of 3–4 m. We monitored the nest boxes each year to determine the hatching date (day the first egg hatched each year = day 0) and brood size at day 13.

During the spring of 2019, we conducted a cross-fostering study to identify the potential effect of nestling characteristics in the 2 habitats on *Culicoides* abundance in their nests. Hence, we designed a cross-fostering experiment in which whole broods were exchanged between the dry and humid forests or within each forest, according to the treatment. When nestlings were 3 days old, broods of the same age were exchanged according to their size (±2 nestlings) using warm, breathable bags. Nestling broods exchanged and reared in the same forest served as manipulation controls. All nestlings in the nest boxes were exchanged, but the procedure was performed in 2 steps to ensure the nests always contained at least 3 nestlings and therefore prevent parent desertion. In total, the cross-fostering experiment included broods from 35 nests: 11 broods were exchanged within the humid forest (humid–humid treatment), 6 within the dry forest (dry–dry treatment), 9 were moved from the dry to the humid forest (dry–humid treatment) and 9 were moved from the humid to the dry forest (humid–dry treatment).

### *Culicoides* collection and identification

Biting midges were captured in blue tit nest boxes following the method described by Tomás *et al*. (2008*b*) with minor modifications. A Petri dish (60 mm diameter) layered with body gel-oil (Johnson's^®^ Baby Oil Gel with Chamomile, Johnson and Johnson, Dusseldorf, Germany) was placed in the inner roof of the nest boxes when nestlings were 12 days old. The gel-oil was made up of paraffinum liquidum, hexyl laurate, ethylene/propylene/styrene copolymer, cyclopentasiloxane, butylene/ethylene/styrene copolymer, chamomilla recutita, bisabolol and perfume (FPT1353). The Petri dishes were collected the next day, when nestlings were 13 days old, and stored in a freezer until further analysis.

The biting midges were removed from the Petri dishes by applying xylene for a few seconds, then passed to absolute ethanol. After approximately 5 min, the biting midges were transferred to Eppendorf tubes with 70% ethanol and maintained at −20°C. The *Culicoides* specimens were sexed and identified to the species level according to their morphological characteristics (e.g. wing spot patterns and the presence of coeloconic sensilla on the antennae) and based on available keys (Rawlings, 1996; González and Goldarazena, 2011), including the IIKC website (Mathieu *et al*., 2012). Species identification was further confirmed by mounting between 4 and 10 individuals of each species. The parity of *Culicoides* females was determined visually: (1) those that had never fed on blood (nulliparous females), (2) those showing a burgundy pigment in the subcutaneous cells of the abdomen indicating a previously digested blood meal (parous females; Dyce, 1969) and (3) those with a recent blood meal in their abdomen (engorged females). We calculated the species richness for each nest box as the sum of the different *Culicoides* species collected. The prevalence of biting midges was calculated as the percentage of infested nests with respect to the total number of nests analysed. We estimated the abundance of *Culicoides* as the number of specimens captured for all the species in each nest. The total abundance of *Culicoides* was calculated as the sum of nulliparous, parous and engorged females per nest, while also considering any unidentified individuals.

### Statistical analyses

We used Cleveland plots to check for outliers in the abundance of biting midges and tested the normality of the abundances of *Culicoides* and species richness graphically (Zuur *et al*., 2010). An outlier was detected in a nest box from the humid forest in 2019, which far exceeded the standard deviation (s.d.) of the mean biting midge abundance (mean ± s.d. = 1.04 ± 1.73; *n* = 94; outlier: 49 individuals). This outlier probably reflected a close breeding area of *Culicoides reconditus*, as 39 out of 49 of the individuals collected corresponded to this species, and 32 of the 39 were nulliparous females. Thus, we performed the analyses using both the original dataset and one that excluded the outlier. Models including the outlier gave qualitatively the same results as those without it. Here, we report the statistical analyses without the outlier, although we included it in the descriptive statistics. The total abundance of *Culicoides* females and species richness followed a Poisson distribution. Analyses on *Culicoides* species were restricted to the 2 most common species captured, namely *C. reconditus* and *Culicoides circumscriptus* (see Table 2).

**Table 2.** Abundance of *Culicoides* species captured in blue tit nests from 2 different types of forests during the breeding seasons of 2017, 2018 and 2019

	2017		2018		2019		Total (*n* = 95)
	Dry forest (*n* = 12)	Humid forest (*n* = 11)	Dry forest (*n* = 18)	Humid forest (*n* = 19)	Dry forest (*n* = 15)	Humid forest (*n* = 20)	
*C. reconditus*	1 (8.33)	2 (18.18)	6 (22.22)	2 (5.26)	7 (33.33)	41 (15.00)	59 (16.84)
*C. circumscriptus*	2 (16.67)	0 (0)	23 (50.00)	8 (26.32)	0 (0)	0 (0)	33 (16.84)
*C. kibunensis*	1 (8.33)	1 (9.09)	1 (5.55)	2 (10.52)	8 (20.00)	11 (25.00)	24 (13.68)
*C. truncorum*	3 (25.00)	2 (18.18)	9 (11.11)	1 (5.26)	2 (13.33)	5 (15.00)	22 (13.68)
*C. festivipennis*	0 (0)	0 (0)	0 (0)	1 (5.26)	2 (13.33)	2 (5.00)	5 (4.21)
Unidentified *Culicoides*	1 (8.33)	0 (0)	1 (5.55)	0 (0)	1 (6.67)	1 (5.00)	4 (4.21)
Total	8 (50.00)	5 (27.27)	40 (55.56)	14 (36.84)	20 (46.67)	60 (45.00)	147 (44.21)

The percentage of infected nests is shown in parentheses.

Generalized linear mixed models (GLMMs) with a Poisson distribution and a logit-link function were used to examine the variation in the abundance of biting midges and species richness with forest type. For data collected in 2017 and 2018, the full models had the following structure: total abundance, abundance of *C. reconditus*, abundance of *C. circumscriptus* and species richness were included as dependent variables in separate models; forest type, year and their interaction were fixed factors; hatching date and day 13 brood size were covariates and nest identity was a random factor. We decided to incorporate hatching date and brood size in the models as previous studies highlighted their importance when interpreting the abundance of biting midges in blue tit nests (Tomás *et al*., 2008*a*; Martínez-de la Puente *et al*., 2009*a*, 2009*b*; Castaño-Vázquez and Merino, 2021). We did not consider the impact of the forest–year interaction on the abundance of *C. circumscriptus* because no specimens were collected in the humid forest in 2017. GLMM with a binomial distribution and a logit-link function was used to test the relationship between the presence/absence of *Culicoides* in nests and the type of forest. The presence/absence of *Culicoides* was used as a dependent variable, and forest, year and forest–year interaction as fixed factors. Hatching date and brood size were introduced as covariates and nest identity as random factor.

Generalized linear model (GLM) with a Poisson distribution and a logit-link function was used to test the effect of the cross-fostering experiment. In this case, total *Culicoides* abundance and species richness were included as the dependent variables in each full model and forest of origin, forest of fostering and their interaction were included as fixed factors, with hatching date and brood size as covariates. A full GLM, with a binomial distribution and a logit-link function, and the same structure as the cross-fostering experiment, was also used to check the relationship between the presence/absence of biting midges and treatments.

In all cases, we applied a model–selection approach to choose the best models of all possibilities derived from the aforementioned full models. To do so, we used Akaike's information criterion (AIC) and selected models within a ΔAIC <2 units (Quinn and Keough, 2002). The parameters were estimated by model averaging all models with a ΔAIC under 2 units (Symonds and Moussalli, 2011). We tested the normality of the residuals from the models graphically following Zuur *et al*. (2010). For the descriptive analyses, we used the Pearson product–moment correlation to examine the correlations between continuous variables. A *t*-test was used to analyse the differences in hatching dates between infected and uninfected nests. The basic statistics are given as mean ± standard error (s.e.). All analyses were performed in software R v 4.0.0 (R Development Core Team, 2020), using the packages ‘lme4’ (Bates *et al*., 2020) and ‘MuMIn’ (Bartoń, 2020).

## Results

A total of 147 female biting midges were captured in 42 of the 95 nests monitored during the 3 years (prevalence 44.21%), corresponding to 5 different species (Table 2). Four biting midges (2.72% of the total captured) were not identified to the species level because they lacked wings or other distinctive structures. A mean of 1.55 ± 0.54 (range: 0–49) biting midges were captured per nest. In nests with biting midges, there was a mean of 1.57 ± 0.13 different species (range: 1–4) per nest. *Culicoides reconditus* (40.14%) and *C. circumscriptus* (22.45%) were the most common species found in blue tit nests (Table 2). Most of the 147 *Culicoides* females captured were nulliparous (75.55%), while we captured 34 parous females (23.13%) and only 2 engorged females (1.36%). Parous females corresponded to the species *C. circumscriptus* (*n* = 14), *C. reconditus* (*n* = 11), *Culicoides truncorum* (*n* = 4), *Culicoides kibunensis* (*n* = 2), *Culicoides festivipennis* (*n* = 1) and 2 parous individuals were not identified to the species level. The engorged females belonged to the species *C. truncorum* and *C. kibunensis*. No males were found in the nests.

### Correlative analyses

**Fig. 1.**
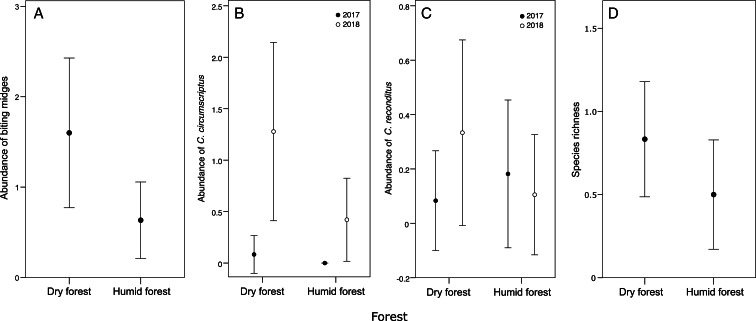
Abundance of biting midges (*Culicoides*) (A), abundance of *Culicoides circumscriptus* (B), abundance of *Culicoides reconditus* (C) and species richness (D) in blue tit nests located in humid and dry forests during the breeding seasons of 2017 and 2018. Means were calculated without the outlier. Bars represent s.e.

**Fig. 2.**
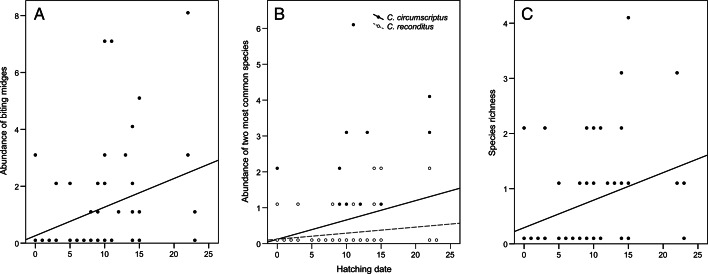
Relationships between hatching date and the abundance of biting midges (*Culicoides*) (A), the abundance of the 2 most common species (*C. reconditus* and *C. circumscriptus*) (B) and species richness (C) in blue tit nests during the breeding seasons of 2017 and 2018. The regression lines were calculated without the outlier (total abundance: adjusted *R*^2^ = 0.084, *P* = 0.014; abundance of *C. reconditus*: *R*^2^ = 0.021, *P* = 0.137; abundance of *C. circumscriptus*: *R*^2^ = 0.055, *P* = 0.039; species richness: adjusted *R*^2^ = 0.081, *P* = 0.016). The hatching date is standardized (0 = day the first egg hatched each year).

Table 3 provides a summary of the results of the model selection for the correlative study. The best model for *Culicoides* abundance included forest and hatching date as predictor variables, while the second-best model also included the year (although it was not significant). The abundance of *Culicoides* captured in blue tit nests was higher in the dry (1.60 ± 0.41) than in the humid forest (0.63 ± 0.21; estimate = 0.99, *z* = 2.31, *P* = 0.021; Fig. 1A) and correlated positively with hatching date (estimate = 0.12, *z* = 3.50, *P* < 0.001; *r* = 0.32, *P* = 0.014; Fig. 2A). The best model for the abundance of *C. circumscriptus* included forest type, year and hatching date as predictors, and the second best also included brood size as a predictor, although it was not significant. The same forest-dependent variation was found for the abundance of *C. circumscriptus* (estimate = 1.25, *z* = 2.07, *P* = 0.039; Fig. 1B), which was higher in 2018 than in 2017 (estimate = 1.94, *z* = 2.37, *P* = 0.018; Fig. 1B). In addition, the abundance of *C. circumscriptus* increased when the nestlings were hatched later in the season (estimate = 0.10, *z* = 1.96, *P* = 0.049; *r* = 0.27, *P* = 0.039; Fig. 2B). The model selected for the abundance of *C. reconditus* included brood size and hatching date as predictors. We found a negative and significant relationship between the abundance of *C. reconditus* and brood size (*χ*^2^ = 8.94, *P* = 0.003; *r* = −0.29, *P* = 0.024). The abundance of *C. reconditus* was positive and significantly related to hatching date (*χ*^2^ = 6.01, *P* = 0.014; Fig. 2B). With respect to species richness, the best model included hatching date and brood size as predictors, but only hatching date was significant. Three additional models had a ΔAIC <2, but only hatching date had a significant effect on species richness in all these models. Specifically, species richness was positively associated with hatching date (estimate = 0.09, *z* = 3.02, *P* = 0.002; *r* = 0.31, *P* = 0.016; Fig. 2C). Finally, the best models for the presence/absence of biting midges included hatching date, forest and year as predictors, but only hatching date significantly affected the presence of *Culicoides* in all these models. The presence of *Culicoides* correlated positively with hatching date (estimate = 0.17, *z* = 2.65, *P* = 0.008), suggesting that nests with late-hatching nestlings were more likely to be infected by biting midges than those which hatch earlier in the breeding season (*t*-test: *t*_58_ = −2.75, *P* = 0.008).

**Table 3.** Models (within ΔAIC <2 units) describing the total abundance of *Culicoides*, species richness, biting midge prevalence and the abundance of the 2 most common *Culicoides* species (*C. reconditus* and *C. circumscriptus*) in blue tit nests during the breeding seasons of 2017 and 2018

Variable	AIC	ΔAIC
Total abundance		
**Forest**, **hatching date**	166.8	0.00
**Forest**, **hatching date**, year	168.2	1.43
Species richness		
**Hatching date**, brood size	130.3	0.00
**Hatching date**, forest	130.5	0.23
**Hatching date**	131.1	0.77
**Hatching date**, forest, brood size	131.3	0.96
Presence/absence		
**Hatching date**, forest	77.0	0.00
**Hatching date**	77.5	0.43
**Hatching date**, year	78.9	1.83
**Hatching date**, year, forest	78.9	1.83
Abundance of *C. reconditus*		
**Hatching date**, **brood size**	55.0	0.00
Abundance of *C. circumscriptus*		
**Forest**, **hatching date**, **year**	106.0	0.00
**Hatching date**, **year**, forest, brood size	107.7	1.75

The significant predictors (*P* < 0.05) are marked in bold.

### Cross-fostering experiment

Table 4 shows the model selection results for the cross-fostering experiment conducted in 2019. The results reflect that the independent variables did not have a significant effect on either species richness or biting midge prevalence as none of the models differed significantly from the null models (Table 4). However, 2 different models for *Culicoides* abundance had a ΔAIC <2 each including hatching date and foster forest (model 1) and hatching date, foster forest and origin forests (model 2) as predictors. However, only hatching date and foster forest had a significant impact on *Culicoides* abundance. As for the correlative study, the abundance of *Culicoides* was higher in the dry (1.33 ± 0.52) than in the humid forest (0.58 ± 0.19; estimate = 0.85, *z* = 2.13, *P* = 0.033) and was positively associated with hatching date (estimate = 0.11, *z* = 2.70, *P* = 0.007).

**Table 4.** Models (within ΔAIC <2 units) describing the total abundance of *Culicoides*, species richness and prevalence of biting midges in blue tit nests during the cross-fostering experiment conducted in 2019

Variable	AIC	ΔAIC
Total abundance		
**Foster**, **hatching date**	96.1	0.00
**Foster**, **hatching date**, origin	97.8	1.69
Species richness		
Null model^a^	75.8	0.00
Presence/absence		
Brood size	48.8	0.00
Null model^a^	48.8	0.03
Hatching date	50.1	1.36

The significant predictors (*P* < 0.05) are marked in bold.

a Null models only include the intercept.

## Discussion

We identified *Culicoides* species composition and examined different factors that influence their abundance in blue tit nests. We found 5 *Culicoides* species, all previously captured in bird nest boxes in other regions (Table 1). In addition, 3 out of 5 *Culicoides* species found here were previously recorded in nests from the same bird species in central Spain, although the dominant species clearly differed between locations (Martínez-de la Puente *et al*., 2009*a*; this study). Specifically, *Culicoides simulator* was the most common species in central Spain accounting for 56.89% of all *Culicoides* captured in blue tit nests, while the most common species observed in the present study was *C. reconditus* (40.14%). Interestingly, to the best of our knowledge, this is the first account of *C. reconditus* in the Iberian Peninsula (Delécolle, 2002; Alarcón-Elbal and Lucientes, 2012). In a previous study in central Spain, Martínez-de la Puente *et al*. (2011*a*) found a high intraspecific genetic variance of a fragment of the cytochrome c oxidase subunit 1 gene in specimens morphologically identified as *Culicoides segnis*, which suggests the sequences could correspond to 2 different species. The authors argued that the results could be due to the presence of *C. reconditus* in the area, because it is closely related to *C. segnis* only differing in the distribution of their coeloconic sensilla on the antennae and shape of abdominal sclerites (Mathieu *et al*., 2012). These characteristics were clearly identified in 10 specimens mounted in this study, 2 of which were deposited in the National Museum of Natural Sciences (MNCN-CSIC), Madrid, Spain, mounted on 4 slides (2 per individual) under the accession numbers MNCN_Ent_319173, MNCN_Ent_267740, MNCN_Ent_267741 and MNCN_Ent_267742.

### Correlative analyses

We found habitat influenced on the abundance of biting midges with a higher abundance in nests located in the dry forest compared to the humid forest over the 2 years of the correlative study. Some of the possible causes of this difference include the interhabitat variation in the total abundance of *Culicoides* and/or because the *Culicoides* have a different capacity to reach avian nests in each habitat. The availability and extension of water sources in a habitat, together with abiotic soil characteristics, are strong determinants of *Culicoides* abundance because of their importance for larval development (Uslu and Dik, 2010; Erram *et al*., 2019). For instance, *C. festivipennis* preferably breeds in nutrient-rich muds found in streams, while other species, such as *C. circumscriptus*, are more generalist when breeding (Uslu and Dik, 2010, and references therein). On the other hand, the weather conditions in the 2 habitats are evidently different; the dry forest had a higher temperature and a lower humidity than the humid forest (Supplementary material; Garrido-Bautista *et al*., 2021). The relative humidity may negatively affect the large-scale abundance of biting midges in the area (van Hoesel *et al*., 2019), which could partially explain the lower *Culicoides* abundance in the humid forest. In addition, in an experimental study affecting the humidity inside nest boxes occupied by European roller (*Coracias garrulus*), Castaño-Vázquez *et al*. (2022) found a lower abundance of *Culicoides* in nests with a higher humidity. Nevertheless, humidity seems to be less determinant for *Culicoides* abundance and flight performance than temperature. During the breeding season of the blue tits, ambient temperature correlates with nest temperature (Ardia *et al*., 2006), while *Culicoides* abundance increases with ambient temperature (Bernotienė *et al*., 2021; Castaño-Vázquez and Merino, 2021) and temperature inside the nest (Martínez-de la Puente *et al*., 2010*b*). In fact, heat gradients are important cues which biting midges, and other vectors, use to locate their hosts (Lehane, 2005).

On the other hand, other variables, such as early morning wind speed – when biting midges are more active – (Lehane, 2005), could affect the number of vectors visiting nest boxes. The dry and humid forests were located opposite each other on south-west and south-east mountain slopes, respectively. Since the prevailing wind in this region is westerly during spring (Viedma-Muñoz, 1998), the humid forest was expected to receive higher wind speeds, ultimately reducing *Culicoides* flight activity and, consequently, decreasing their abundance in bird nests (Martínez-de la Puente *et al*., 2009*b*). Variations in forest leaf density between large areas may also impact *Culicoides* population numbers, with some species favouring sparsely vegetated areas, while others prefer habitats with a higher leaf density (Conte *et al*., 2007). This variation in forest cover goes some way to explaining the different abundances of some *Culicoides* species between habitats. Lastly, we should not ignore the fact that the interforest variation in *Culicoides* abundance could be due to a geographical singularity of the 2 sampled localities independent of the habitat differences; however, unfortunately, we cannot study this premise as we do not have any spatial replicates for this system.

Differences in the weather conditions may also explain the positive association between hatching date and all the variables analysed. This implies that nestlings from nests breeding later in the season were affected by more *Culicoides* and from more species. As the ambient temperature increases throughout the breeding season, we would normally expect more biting midges to visit more nests (e.g. Bernotienė *et al*., 2021). Several studies have reported effects of seasonality on the abundance of different vector groups, including *Culicoides*, and found that their abundance generally augmented as the spring progressed (Sarto i Monteys and Saiz-Ardanaz, 2003; Ferraguti *et al*., 2013; Lalubin *et al*., 2013; Bernotienė *et al*., 2021). This was also true of cavity-nesting birds, as *Culicoides* abundance in their nests increased as the breeding season advanced (Tomás *et al*., 2008*a*; Martínez-de la Puente *et al*., 2009*a*, 2009*b*; Castaño-Vázquez and Merino, 2021; this study). In addition to the detrimental effect of the blood-sucking activity of *Culicoides*, these results support the fact that nestlings which hatch later in the season may be subject to a greater susceptibility to blood parasite infections (Martínez-de la Puente *et al*., 2013). At least, 4 out of 5 species of *Culicoides* captured here may act as vectors for *Haemoproteus* parasites, and parous females of all of these species have been found in avian nests (*C. circumscriptus*: Martínez-de la Puente *et al*., 2011*a*; Veiga *et al*., 2018; *C. festivipennis*: Martínez-de la Puente *et al*., 2011*a*; *C. kibunensis*: Martínez-de la Puente *et al*., 2011*a*; Bernotienė *et al*., 2019; Žiegytė *et al*., 2021; *C. truncorum*: Martínez-de la Puente *et al*., 2011*a*). Thus, nestlings from late-nesting parents could be impaired in terms of future reproduction success (Merino *et al*., 2000) or even long-term survival (Martínez-de la Puente *et al*., 2010*a*). Further studies should be conducted in order to identify the parasites potentially transmitted by *Culicoides* species in the area.

Furthermore, there was a negative relationship between insect abundance and brood size in the case of *C. reconditus*, while the other variables analysed returned statistically non-significant associations. If more nestlings release a greater concentration of attractive molecules (e.g. CO_2_, kairomones), then one would expect higher *Culicoides* abundances in nests with larger broods (Martínez-de la Puente *et al*., 2009*a*, 2010*b*; Castaño-Vázquez and Merino, 2021). Nevertheless, contrasting results have previously been reported in blue tits, with some studies showing a positive relationship between *Culicoides* abundance and brood size (Martínez-de la Puente *et al*., 2009*a*, 2009*b*), yet another study also reported non-significant associations (Tomás *et al*., 2008*a*). Given the correlative nature of these results, further experimental research into different brood sizes is required to clarify the influence of brood size on the birds' susceptibility to *Culicoides* attacks.

### Cross-fostering experiment

Based on the results from the previous 2 years, we developed an experimental approach to identify the role of habitat *vs* nestling traits in determining the birds' susceptibility to *Culicoides* attacks. Nestlings from different habitats could produce different attractants to insect vectors. For example, different studies have proposed that odours derived from avian uropygial gland secretions are involved in the attraction of different vectors (Russell and Hunter, 2005; Garvin *et al*., 2018; review in Moreno-Rueda, 2017; Tomás *et al*., 2020). This secretion, together with other scent-producing body sources, such as the skin or feathers (Menon and Menon, 2000; Campagna *et al*., 2012), may determine bird odour (Campagna *et al*., 2012) and probably has a genetic origin (Krause *et al*., 2018). For the case of biting midges, Tomás *et al*. (2020) found that uropygial secretions from hoopoe (*Upupa epops*) nestlings may repel some insect vectors, depending on the habitat. However, other studies failed to identify any similar associations in biting midges (Martínez-de la Puente *et al*., 2011*b*) and mosquitos (Díez-Fernández *et al*., 2019). In addition, uropygial secretions can harbour symbiotic bacteria that may release several chemical cues (Maraci *et al*., 2018), which, together with the skin microbiota, may affect vector attraction (reviewed in Ruiz-López, 2020). However, in this work we failed to identify any significant association supporting this scenario, which suggests that habitat may have a major significant impact on nestling exposure to biting midges. Our results indicate that the habitat of origin of blue tit nestlings did not affect the degree to which they attracted biting midges to their nests, suggesting that nestlings did not exhibit repellent or attractant chemical properties to these vectors unrelated to rearing habitat (due to genetic differentiation or maternal effects). On the other hand, the abundance of biting midges did differ between habitats, lending further support to the influence of this variable on *Culicoides* abundance in avian nests.

## Concluding remarks

The abundance of biting midges in blue tit nests is mainly determined by habitat type, which may explain the different patterns of blood–parasite transmission observed in birds from different habitats (e.g. Ferraguti *et al*., 2018). It is important to take these results into account when trying to understand local variations in bird species' susceptibility to vector attacks. These attacks may adversely affect the health and fitness of wild birds due to the detrimental effects of their bites and the parasites they can transmit. This should be especially relevant due to the impact of global change on the distribution and abundance of vectors of pathogens, including those attacking birds in their nests (Castaño-Vázquez and Merino, 2021).

## Data Availability

Data will be provided by the authors under reasonable request.

## Supplementary material

For supplementary material accompanying this paper visit https://doi.org/10.1017/S003118202200066X. click here to view supplementary material
