# Supplementary material for: Habitat-dependent Culicoides species composition and abundance in blue tit (Cyanistes caeruleus) nests
Source: Parasitology. 2022 May 16;149(8):1119–28. doi: 10.1017/S003118202200066X (PMC10090578; doi:10.1017/S003118202200066X)
Supplement: Supplementary file 1 [file S003118202200066Xsup001.docx]

**Supplementary material**

**From: Habitat-dependent *Culicoides* species composition and abundance in blue tit (*Cyanistes caeruleus*) nests**

**Table S1.** Temperature, relative humidity, irradiation, insolation and NDVI for the two forests of the study area. Table shows the mean values, standard deviation, statistic and p-value.

| **Factor** | **Forest** | | **Statistic** | **p-value** |
| --- | --- | --- | --- | --- |
|  | Dry forest | Humid forest |  |  |
| Temperature (ºC) ^1^ | 17.81 ± 6.47 | 16.80 ± 6.19 | t_1338_ = 14.11 | < 0.001 |
| Relative humidity (%) ^1^ | 41.37 ± 18.02 | 42.99 ± 17.90 | t_1338_ = 9.21 | < 0.001 |
| Insolation (h) ^2^ | 13.46 ± 0.34 | 11.98 ± 0.21 | U = 0.00 | < 0.001 |
| Irradiation (kWh/m^2^/day) ^3^ | 6.63 ± 0.20 | 6.45 ± 0.19 | U = 530.00 | < 0.001 |
| NDVI ^4^ | 0.54 ± 0.08 | 0.59 ± 0.04 | U = 17.00 | 0.112 |

^1^ Data obtained from dataloggers iButton® installed in outer walls of nest-boxes. Year: 2019. Statistic: t-test.
^2^ Data obtained from software GRASS SIG 7.8.2, integrated in QGIS 3.10.5, using the “r.sun.insoltime” algorithm. Insolation obtained every five days and covering breeding seasons of blue tits from 2017 to 2019. Statistic: U-Mann Whitney test.
^3^ Data obtained from software ArGis Desktop 10.3.1, using the “Area solar radiation” algorithm. Solar irradiation obtained every five days for breeding seasons of each year (2017, 2018 and 2019). Statistic: U-Mann Whitney test.
^4^ Data obtained from software QGIS 3.10.5 using Landsat-8 satellite images covering the breeding seasons of each year (2017, 2018 and 2019). NDVI calculated based on Landsat-8 bands B4 (red) and B5 (near infrared) as: (B5 – B4) / (B5 + B4). Statistic: U-Mann Whitney test.
